# Deep vein thrombosis and pulmonary embolism – Prevention, management, and anaesthetic considerations

**DOI:** 10.4103/0019-5049.60490

**Published:** 2010

**Authors:** Krishan Kumar Narani

**Affiliations:** Department of Anaesthesiology, Pain and Perioperative Medicine, Sir Ganga Ram Hospital, New Delhi - 110 060, India

**Keywords:** Anaesthetic considerations, deep vein thrombosis, pulmonary embolism, thromboprophylaxis, venous thromboembolism

## Abstract

There is high incidence of venous thromboembolism, comprising of deep vein thrombosis and pulmonary embolism, in hospitalized patients. The need for systemic thromboprophylaxis is essential, especially in patients with inherited or acquired patient-specific risk factors or in patients undergoing surgeries associated with high incidence of postoperative deep vein thrombosis and pulmonary embolism. These patients, on prophylactic or therapeutic doses of anticoagulants, may present for surgery. General or regional anaesthesia may be considered depending on the type and urgency of surgery and degree of anticoagulation as judged by investigations. The dilemma regarding the type of anaesthesia can be solved if the anaesthesiologist is aware of the pharmacokinetics of drugs affecting haemostasis. The anaesthesiologist must keep abreast with the latest developments of methods and drugs used in the prevention and management of venous thromboembolism and their implications in the conduct of anaesthesia.

## INTRODUCTION

The anaesthesiologists of today, specializing as perioperative physicians, have to often face circumstances (high-risk patients or surgeries) associated with life-threatening conditions. Venous thromboembolism, comprising of deep vein thrombosis and pulmonary embolism, is one such condition. Though the physicians and surgeons in India have started realizing the importance of thromboprophylaxis to prevent morbidity and mortality associated with venous thromboembolism (VTE), there is a widespread variation in the management of this dreaded condition.

Anaesthesiologists must update their knowledge regarding the drugs and methods used in the prevention and management of VTE, as well as their implications in the conduct of anaesthesia.

## INCIDENCE

A 25-year population-based study reported an annual incidence of symptomatic VTE as 117 per 100,000 persons in 1998.[1] The incidence of deep vein thrombosis (DVT) in hospitalized patients has increased from 0.8% to 1.3% over a period of 20 years (reported in 2005).[2] A high incidence of DVT has been found in patients undergoing surgical procedures. Without thromboprophylaxis the incidence of DVT is about 14% in gynaecological surgery, 22% in neurosurgery, 26% in abdominal surgery and 45%-60% in patients undergoing hip and knee surgeries.[3] Though the exact incidence of VTE in the Indian population is not known because of nonuniform reporting of such incidents, its incidence is not expected to be different from that in the western population.[4]

## NATURAL COURSE

DVT in the lower extremity may arise in the calf veins or in the proximal veins. The thrombus may extend proximally to iliac veins and inferior vena cava. The incidence of DVT in the upper extremity is also increasing because of widespread use of central venous catheters. DVT may occur in deep pelvic veins or renal veins. There may be formation of thrombus in the right side of the heart due to atrial fibrillation.

The most clinically important and fatal pulmonary embolism (PE) occurs from proximal than distal DVT in the leg. PE occurs in 50% of patients with proximal DVT,[5] while asymptomatic thrombosis of the leg veins has been observed in 70% of patients with PE.[6]

On early ambulation, the thrombus in the deep veins may resolve completely.

Post-thrombotic syndrome may develop in 25% of patients, 2 years after the initial diagnosis and proper treatment of DVT.[7] The damage to the venous valves causes chronic venous congestion.

Inadequate treatment of DVT results in 20%-50% risk of recurrent VTE,[8] and collaterals develop parallel to the thrombosed segment of the vein.

A chronic unresolved thrombus leads to chronic thromboembolic pulmonary hypertension and right heart failure in 3.8% of patients at 2 years after diagnosis and proper treatment.[9]

## AETIOLOGY[4,10–15]

Rudolph Virchow in 1856 described the factors that predispose to DVT, which are relevant even today. Virchow's triad comprises of 3 factors: venous stasis, damage to venous wall and hypercoagulability.

### Patient-specific risk factors

These are either acquired or inherited hypercoagulable states associated with a high incidence of DVT and PE.

### Inherited patient-specific factors

Factor V Leiden and Cambridge mutation (activated protein C resistance)

Prothrombin gene mutation (20210A)

Congenital deficiencies of Antithrombin III, Protein C, and Protein S

Dysfibrogenaemia

Hyperhomocysteinaemia

An inherited abnormality may not be found in 40%-60% of patients with idiopathic VTE.

### Acquired patient-specific factors

Past history of thromboembolism, malignancy; age>40 years; obesity; varicose veins; prolonged immobilization; dehydration; heart failure; nephrotic syndrome; stroke; myeloproliferative syndrome; pregnancy; puerperium; oral contraceptives; hormone replacement therapy; and antiphospholipid antibody syndrome.

The overall risk of thrombosis in cancer patients is sevenfold as compared to non-cancer patients. Drugs used in cancer may directly contribute to thrombosis[14]

### Surgery-specific risk factors

There are some operative procedures and medical conditions associated with high incidence of postoperative DVT and PE [Tables 1 and 2].[12,13] Depending on the clinical risk factors, patients may be categorized into low-, medium- and high-risk groups.

**Table 1:** Predisposing factors for the development of DVT after surgery[12,13]

Event	Low risk	Moderate risk	High risk
General surgery			
Age (years)	<40	>40	>40
Duration of surgery (Minutes)	<60	>60	>60
Orthopaedic surgery			THR, TKR
Trauma			Extensive soft tissue injury, major fractures, multiple trauma sites
Medical conditions	Pregnancy	Postpartum period MI, CHF	Stroke

DVT: Deep vein thrombosis,

**Table 2:** Incidence of DVT and PE after surgery[12,13]

Event	Low risk %	Moderate risk %	High risk %
DVT without prophylaxis	2	10-40	40-80
Symptomatic PE	0.2	1-8	5-10
Fatal PE	0.002	0.1-0.4	1-5

DVT: Deep vein thrombosis, PE: Pulmonary embolism

## SIGNS AND SYMPTOMS OF DVT[4,10,12]

The clinical diagnosis is difficult as the signs and symptoms are not specific. Some patients may complain of pain in the calf muscles and thighs and may present with swollen legs. There may be presence of tenderness, a palpable thickened vein, distended veins, discolouration or cyanosis.

## DIAGNOSIS OF DVT[4,10,12,16–18]

1. Compression/duplex ultrasonography of femoral and popliteal veins has both sensitivity and specificity of 97% in detecting DVT in a symptomatic patient. It is sensitive for proximal vein thrombosis but less sensitive for calf vein thrombosis.
2. Impedance plethysmography records the electrical impedance of the calf region following a temporary occlusion of proximal veins. The sensitivity of this method is 96%, 50% and 38% for the diagnosis of acute DVT of the proximal, popliteal and distal veins, respectively.
3. Contrast venography remains the gold standard for the diagnosis of DVT. It is able to detect all clinical forms of DVT, including thrombosis in calf veins, pelvis and inferior vena cava.
4. Radionuclide ascending venography assesses the “thrombus burden” in the femoral, iliac, caval and pulmonary circulation.[16] It has a sensitivity of 90% and specificity of 92% in detecting DVT in the proximal leg veins.
5. Plasma D-dimer is a marker of cross-linked fibrin degradation products. A negative D-dimer result can exclude DVT and PE in a patient with suspected VTE.[17,18]

Once VTE is diagnosed, further tests may be done:

1. To have baseline tests — prothrombin time (PT), activated partial thromboplastin time (APTT) and platelet count, before starting anticoagulants.
2. To identify inherited risk factors as the cause of VTE, as these patients require lifelong or extended period of management. In acute life-saving situations, this may not be possible.

## PULMONARY EMBOLISM[4,19–20]

The clinical diagnosis of acute pulmonary embolism is not very accurate as signs and symptoms of PE are not very specific. Based on the clinical presentation, Well's Diagnostic Scoring System has been used for the diagnosis of PE. A high probability score, viz., >6 out of a maximum score of 12.5, represents high probability (58%) of PE. The diagnosis becomes more difficult in patients with coexisting cardiac or pulmonary disease. The most frequent clinical manifestations in patients with PE are dyspnoea, tachypnoea, pleuritic pain, one or more of which occur in 97% of patients with PE.[21]

## DIAGNOSIS OF PE

Certain diagnostic tests are performed to confirm the diagnosis of PE.

1. ECG — An S wave in lead I, a Q wave in lead III, an inverted T wave in lead III are some of the characteristic changes in acute PE (S_1_, Q_3_, T_3_ pattern).[19,21] Arrhythmias, right ventricular strain, P pulmonale, right ventricular hypertrophy, and right bundle branch block may be present in some cases. Non-specific abnormalities of the ST segment or T wave may occur in more than 49% of cases.[21]
2. X-ray chest — Atelectasis or pulmonary parenchymal abnormality may occur in 68% of patients. An elevated haemidiaphragm, pleural effusion or pulmonary oedema may be present[19–21]
3. Arterial blood gas analysis (ABG) analysis — PaO_2_ of ≤80 mm Hg may be present in 26% of patients.[19,21]
4. Ventilation/perfusion scan (V/Q scan)[22–28] — Interpretation of V/Q scan, using PIOPED criteria (National Collaborative Study of the Prospective Investigation of Pulmonary Embolism Diagnosis) showed high-probability results indicative of PE in 87% of patients. A normal V/Q scan entirely excluded PE.
5. Pulmonary angiography is a gold standard test, but less frequently performed due to the availability of CT/MRI/spiral CT angiography. A diagnostic strategy that includes clinical evaluation, V/Q scan and evaluation for DVT would decrease the number of patients who require pulmonary angiography from 72% to 33%.[28]
6. CT/MRI/spiral CT angiography has a high specificity for the identification of main and lobar emboli and can exclude other pulmonary diseases.
7. Plasma D-dimer[18,19] — A negative D-dimer result can exclude DVT and PE in a patient with suspected VTE.
8. Detection of proximal DVT[29–31] — Confirms the diagnosis in patients with suspected PE.

## MANAGEMENT OF DVT AND PE[4,10,12,31–33]

The initiation of appropriate therapy with anticoagulants is as important as prompt and accurate diagnosis to decrease the incidence of potentially fatal complications.

1. Unfractionated heparin (UFH) initial bolus dose 80 IU/kg or 5000 IU IV followed by continuous infusion at a dose of 18 IU/kg/h is started. Dose is adjusted to maintain APTT at 1.5-2.5 times the control value. Alternatively, subcutaneous low-molecular weight heparin (LMWH) or direct anti-Xa inhibitor (fondaparinux) may be given [Table 3].

**Table 3:** Therapeutic dosage of various LMWHs and direct Xa inhibitor[10]

LMWH	
Dalteparin	200 IU/kg s.c. od, 150 IU/kg s.c. od after 1 month
Enoxaparin	100 IU/kg s.c. bid or 150 IU/kg s.c. od
Tinzaparin	175 IU/kg od
Nadroparin	4100 IU s.c. bid (<50 kg)
	6150 IU s.c. bid (50-70 kg)
	9200 IU s.c. bid (>70 kg)
Parnaparin	6400 IU s.c. bid
Reviparin	3500 IU s.c. bid (35-45 kg)
	4200 IU s.c. bid (40-60 kg)
	6300 IU s.c. bid (>60 kg)
Direct Xa Inhibitor	
Fondaparinux	5 mg s.c. od (<50 kg)
	7.5 mg s.c. od (50 – 100 kg)
	10 mg s.c. od (>100 kg)

LMWH: Low–molecular weight heparin
2. Oral vitamin K antagonist (VKA) is started within 24 hours of heparin therapy, and dose is adjusted to maintain a target International normalized ratio (INR) of 2.5 (INR range, 2.0-3.0).
3. Heparin/LMWH/Fondaparinux are discontinued when VKA has been given for 5 days and INR is >2.0 for 2 consecutive days for DVT (INR >3.0 for 2 consecutive days for PE).
4. VKA is continued for at least 3 months in patients with the first episode of VTE due to a transient, reversible risk factor. However, in patients with inherited thrombophilia, it should be continued for at least 6-12 months after the first episode of VTE. In patients with a second or recurrent episode, it should be continued indefinitely.
5. Thrombolytic treatment (catheter directed or systemic) with urokinase, streptokinase, tissue plasminogen activator may be tried in patients with extensive acute proximal DVT (symptoms, <14 days; life expectancy, >1 year) or PE associated with hypotension, cardiogenic shock or right ventricular failure. Thrombolytic treatment is followed by anticoagulation.
6. Operative venous thrombectomy may be considered in patients with acute DVT (symptoms, <7 days; life expectancy, >1 year).
7. Ligation or partial occlusion of inferior vena cava (IVC) is an outdated method of management of VTE.
8. Inferior vena cava filter (IVC filter)[4,31–32,34–36] is indicated in patients having complications or contraindications to anticoagulant therapy. However, there is an increased incidence of recurrent DVT 1-2 years after insertion of IVC filter. To offset this problem, a retrievable IVC filter may be used. Long-term therapy with anticoagulants, direct thrombin or anti-Xa inhibitors should be restarted as soon as possible.
9. Pulmonary embolectomy may be considered in highly compromised patients to relieve acute obstruction as a result of massive PE or in patients in whom thrombolytic treatment is contraindicated because of bleeding risks. This procedure is associated with high mortality.[31,32]
10. Pulmonary thrombo-endarterectomy may be considered in patients with recurrent PE leading to chronic thromboembolic pulmonary hypertension and right heart failure.[32,37]

## THROMBOPROPHYLAXIS OF VTE[4,11,15,38,39]

There is a high incidence of VTE in hospitalized patients, particularly in patients with high-risk factors (acquired or inherited thrombophilia) or in patients undergoing high-risk surgeries. In the management of surgical patients, early ambulation, mechanical and/or pharmacologic thromboprophylaxis have been found to decrease the incidence of DVT and fatal PE. As VTE is an important health-care problem not only in the western world but also in the developing countries like India, the importance of thromboprophylaxis in the Indian population cannot be overemphasized.

Early ambulation[4,11,15,38,39] in the postoperative period should be encouraged for all patients. In patients having no additional risk factors and in those undergoing low-risk surgical procedures, early ambulation may be the only method of thromboprophylaxis.

## MECHANICAL THROMBOPROPHYLAXIS

Mechanical methods may be used alone or along with pharmacological thromboprophylaxis. These devices augment venous blood flow and thus prevent venous stasis in the leg veins. They are contraindicated in patients with established DVT.

Graduated compression stockings apply pressure of varying degree to the leg and thigh, the pressure being greatest at the ankle and decreasing proximally. The pressure gradient is able to prevent venous stasis.

Intermittent pneumatic compression is applied to the legs. These cuffs inflate and deflate alternately to prevent venous stasis.

Mechanical foot pumps help in intermittent plantar compression (IPC) in each foot and augment blood flow in the leg veins.

## PHARMACOLOGIC THROMBOPROPHYLAXIS[4,11,15,38–39]

The various drugs used are as follows:

### Unfractionated heparin

For moderate-risk patients, UFH subcutaneous (s.c.) 5000 IU bid; and for high-risk patients, s.c. 5000 IU tid or 7500 IU bid, with the first dose given 2 hours preoperatively. APTT >1.5 times the control value provides adequate thromboprophylaxis.

There is an increased incidence of major bleeding in 5% of cases.

A prothrombotic immune-mediated, heparin-induced thrombocytopenia (HIT)[40] occurs in 0.5% to 5% of patients treated for at least 5 days with UFH. Antibodies to heparin-PF4 complex are formed. HIT is characterized by an unexplained 50% or more decrease in the platelet count, often to <150 × 10^9^/L. Heparin should be discontinued in such situation.

### Low-molecular weight heparins

LMWHs have demonstrated their efficacy and safety as drugs of choice in the prophylaxis of thromboembolism. LMWHs produce therapeutic effect within 2 hours, and peak plasma level is achieved within 4 hours. This decreases to 50% of peak values 12 hours after subcutaneous injection.[41] LMWHs cause antithrombin-mediated inactivation, mainly of FXa and less of FIIa. Lab assessment of anti-Xa is routinely not required. Therapeutic range of anti-Xa is 0.5-1.0 units/mL. APTT may be prolonged when anti-Xa is >1.0 units/mL. R time on thrombelastography correlates with anti-Xa concentration.[42]

Table 4 compares mean molecular weight and anti-Xa to anti-IIa activity of unfractionated heparin with different LMWHs.

**Table 4:** Mean mol. wt. and anti Xa to IIa ratio of unfractionated heparin and LMWHs[41]

Type of heparin	Mean mol. wt. (Daltons)	Anti-Xa to IIa-ratio
Unfractionated heparin	13000	1.0
Tinzaparin	5500	1.5
Parnaparin	4500	2.4
Dalteparin	5000	2.5
Nadroparin	4500	3.2
Enoxaparin	4400	3.9
Reviparin	3900	4.1

There are three prophylactic LMWH regimens in use in patients undergoing high-risk surgeries:

1. Prophylaxis started 12 hours before surgery (followed in European countries).
2. Prophylaxis started 12-24 hours after surgery (followed in North America).
3. Prophylaxis is started more than 12 hours before or 12 hours after surgery (followed by physicians who do not follow above regimens).

For indication and dosage, the physician should refer to the hospital's policies for the prevention of VTE and product inserts for further details.

### Vitamin K antagonist

Warfarin sulfate or nicoumalone (acitrom) is started on the day of surgery, either preoperatively; or postoperatively in the evening. It is monitored by prothrombin time (PT) (target INR, 2.5; range, 2-3).

### Direct FXa inhibitors (Fondaparinux)

It is administered in the dose of 2.5 mg s.c. once daily and is started 6-8 hours after surgery or next day after surgery.

### Antiplatelet drugs (Aspirin)[43,44]

There has been a resurgence of interest in the use of low-dose aspirin for thromboprophylaxis of VTE; however, aspirin should be used as an adjunct to either UFH or LMWH.

## THROMBOPROPHYLAXIS FOR SURGICAL PROCEDURES[4,11,15,38,39]

1. Wherever possible, early ambulation should be encouraged in the postoperative period.
2. Mechanical methods of thromboprophylaxis may be used primarily in patients at high risk of bleeding or as an adjunct to anticoagulant-based thromboprophylaxis.
3. Thromboprophylaxis with UFH, LMWH or fondaparinux should be considered for all patients undergoing major general surgery, gynaecologic surgery and open urologic surgery.
4. For hip- or knee-replacement surgery (THR or TKR), LMWH, fondaparinux or VKA may be preferred.
  For THR, LMWH is started 12 hours before surgery; or 12-24 hours after surgery; or 4-6 hours after surgery at half of the dose, and full dose on the following day.
  Fondaparinux 2.5 mg is started 6-8 hours after surgery or next day after surgery.
  VKA is started preoperatively or in the evening of the surgery day (target INR, 2.5; range, 2-3).
  For patients with a high risk of bleeding undergoing THR or hip-fracture surgery, mechanical thromboprophylaxis may be considered. When the high risk of bleeding decreases, pharmacologic thromboprophylaxis may be started.
5. For TKR, intermittent pneumatic compression and/or routine thromboprophylaxis with LMWH, fondaparinux or warfarin may be used (INR, 2-3). Low-dose aspirin or UFH as the only method of thromboprophylaxis is not recommended. In patients with high risk of bleeding, mechanical thromboprophylaxis with Intermittent pneumatic compression (IPC) may be used. When the high risk of bleeding decreases, pharmacologic thromboprophylaxis may be added. Thromboprophylaxis should be continued for 10 days or up to 35 days after surgery.
6. For major neurosurgical procedures, the main concern is the risk of bleeding in the perioperative period. So mechanical prophylaxis may be preferred during surgery. In addition, LMWH may be prescribed in the postoperative period in very high risk cases. The decision regarding the use of LMWH should be individualized based on the potential risk of bleeding.
7. The benefit/risk of prophylaxis/bleeding should be individualized in all surgical procedures.

## ANAESTHETIC MANAGEMENT

Two groups of patients may present for surgery:

1. Patients with no past history of venous thromboembolism, posted for prolonged or high-risk surgery.
2. Patients with definite past history of venous thromboembolism (DVT/PE) with or without IVC filter.

## PREOPERATIVE PREPARATION

History of associated co-morbid conditions, with special reference to the risk factors should be noted. A thorough physical examination is mandatory. Details of anticoagulant drugs — name, type, dosage, duration of treatment, timing of the last dose and duration of discontinuation of the drug — should be noted. Risk/benefit of discontinuation of anticoagulants should be explained to the patient. An informed consent stating the risks involved in the perioperative period should be taken.

## PREOPERATIVE INVESTIGATIONS

1. Bleeding time, platelet count, PT and APTT may be performed on the day of surgery.
2. Assessment of anti-Xa level is routinely not required but may be requested in selected cases[42]
3. Thromboelastography may be performed since its parameters have been found to be better predictors of perioperative bleeding.[45]
4. Coagu Chek XS Plus (Roche Diagnostics) — A small, hand-held instrument monitors PT and INR and is useful in an emergency situation[46,47]

## INTRAOPERATIVE MONITORING

Pulse, Non-invasive blood pressure (NIBP), SpO_2_, EtCO_2_, ECG and ST analysis are sufficient in most of the cases.

In high-risk patients, Central venous pressure (CVP) and arterial BP may be considered.

ABG analysis and transoesophageal echocardiography may be helpful in suspected cases of PE.

## ANAESTHETIC CONSIDERATIONS

As the number of patients being treated with drugs interfering with haemostasis is increasing, the anaesthesiologist is faced with a dilemma in administering anaesthesia to such patients. General or regional anaesthesiologist may be considered depending on the type of surgery, urgency of surgery and degree of anticoagulation as judged by investigations.

### General anaesthesia

If general anaesthesia is planned, then balanced anaesthesia as for any major surgery may be administered. The co-morbid conditions require special attention during anaesthesia.

In selective cases, compression stockings or intermittent pneumatic compression may be used on the lower limbs to prevent DVT.

There is marked increase in tissue factor, vWF, plasminogen activator inhibitor-1 (PAI-1) and tissue plasminogen activator, resulting in a hypercoagulable and hypofibrinolytic state postoperatively in patients receiving general anaesthesia.[48,49] This has been demonstrated by increased levels of thrombin-antithrombin complexes and fibrinopeptide A. PAI-1 levels in patients receiving epidural anaesthesia remain normal at preoperative level. Therefore, epidural anaesthesia has been found to be helpful in preventing hypercoagulable state and DVT.[50,51] Surgical procedures and other factors such as immobility, infections, malignancy, drugs, hypothermia, metabolic acidosis, colloids and extracorporeal circulation disturb the fine balance of haemostatic system.

Meticulous intraoperative monitoring is important to diagnose pulmonary embolism during surgery.

### Regional anaesthesia[11,52]

The American Society of Regional Anaesthesia guidelines on regional anaesthesia in patients on various anticoagulant agents may be followed if regional anaesthesia is planned.

### Heparin

1. Epidural needle/catheter placement/removal may be performed 2 to 4 hours after the last heparin dose if APTT is <1.5 times the normal values.
2. Heparin administration should be delayed for 1 hour after the placement of epidural needle or catheter.

### LMWH

1. Epidural needle/catheter placement/removal may be performed 10 to 12 hours after the last thromboprophylactic dose and 24 hours after the last therapeutic dose of LMWH.
2. Avoid neuraxial techniques if patient has received LMWH dose 2 hours preoperatively as this coincides with peak anticoagulant activity.

### Vitamin K antagonist

1. Discontinue VKA 4-5 days before neuraxial block (INR should be <1.2).[11,53,54]
2. In high-risk patients, “no anticoagulation period” should be kept as short as possible. After discontinuation of these drugs, a high-risk patient may be switched over to heparin or LMWH.

### Thrombolytic drugs

Avoid central neuraxial block after thrombolytics therapy with streptokinase, urokinase and recombinant tissue plasminogen activator.

### Direct thrombin inhibitors or anti-Xa drugs

Fondaparinux (Arixtra) — avoid central neuraxial block.

Melagatran, Ximelagatran — should be stopped 12 hours before surgery.

### Antiplatelet drugs

1. Low-dose aspirin (up to 75 mg) does not pose an added risk when performing a neuraxial block and can be continued till the day of surgery.
2. Normally, other antiplatelet drugs are not prescribed either for the thromboprophylaxis or for the treatment of VTE. It may not be possible to discontinue antiplatelet drugs in patients with unstable angina or drug-eluting coronary stents. One must forgo the benefits of regional anaesthesia in such patients and consider general anaesthesia. Discontinue clopidogrel for 7 days and ticlopidine for 14 days before neuraxial block.
3. Epidural needle/catheter placement/removal can be performed 1 to 2 days after the last dose of platelet GP IIb/IIIa inhibitors (abciximab, eptifibatide, tirofiban).

### When blood trickles out of epidural or spinal needle in such patients[52]

The procedure should be abandoned and the patient should be monitored for any neurologic dysfunction. Some of the workers do not cancel the procedure as there is no clinical data to support this recommendation. In this situation, the initiation of LMWH therapy should be delayed for 24 hours postoperatively.

### Non-neuraxial blocks (plexus and peripheral nerve blocks)[52]

There are no definitive recommendations, and the same guidelines as for neuraxial block may also be applied to plexus and peripheral nerve blocks.

## POSTOPERATIVE MANAGEMENT

Monitor postoperatively for signs and symptoms of spinal cord compression as a result of spinal/epidural haematoma. These may present as progression of sensory or motor block, bowel or bladder dysfunction[55] The interval of neurologic monitoring should not be more than 2 hours.

Decompression laminectomy should be performed as soon as the diagnosis of spinal haematoma is confirmed, preferably within 8 hours.

To keep the “no anticoagulation period” as short as possible, it is advisable to restart the anticoagulants after surgery.

## EMERGENCY SURGERY

In an emergency situation, there may not be time to normalize the coagulation profile before surgery. Stopping these drugs before surgery is not the option in any emergency surgery as it takes days to normalize the coagulation profile.

On stopping warfarin before surgery, spontaneous normalization of the INR takes about 4 days if INR is between 2 and 3.[53] Vitamin K takes at least 24 hours to fully antagonize the effect of warfarin. The effect of therapeutic doses of tinzaparin (LMWH) may last for 24 hours.[56]

Protamine sulfate may be used in equimolar dose to reverse the effect of unfractionated heparin.

There are no recommendations for the use of protamine sulfate for the reversal of LMWH, though 1 mg of protamine sulfate for every 100 anti-Xa units of LMWH may reverse more than 90% of the anti-IIa and 60% of anti-Xa activity. Both anti-IIa activity and anti-Xa activity may return up to 3 hours after protamine reversal, possibly due to release of additional LMWH from the subcutaneous depot.[41]

Fresh frozen plasma, prothrombin complex concentrate may be used to normalize INR within minutes. The recombinant-activated FVIIa can also normalize INR quickly.[57]

## EMERGING TRENDS

Orally administered direct thrombin inhibitor (Dabigatran) and direct FXa inhibitor (Rivaroxaban) have the potential to simplify long-term anticoagulant therapy.

## CONCLUSION

Deep vein thrombosis followed by fatal pulmonary embolism is common in certain high-risk patients and after some high-risk surgical procedures. Because of high mortality, prophylaxis against venous thromboembolism has attained widespread acceptance. These patients, on prophylactic or therapeutic doses of anticoagulants, may present for surgery.

The dilemma regarding general anaesthesia versus regional anaesthesia can be resolved if the anaesthesiologist is aware of the pharmacokinetics of anticoagulant drugs. Correct diagnosis and prompt management of thromboembolic events in the perioperative period reduces mortality in such patients.
